# NbX: Machine Learning-Guided Re-Ranking of Nanobody–Antigen Binding Poses

**DOI:** 10.3390/ph14100968

**Published:** 2021-09-24

**Authors:** Chunlai Tam, Ashutosh Kumar, Kam Y. J. Zhang

**Affiliations:** 1 Laboratory for Structural Bioinformatics, Center for Biosystems Dynamics Research, RIKEN, 1-7-22 Suehiro, Tsurumi, Yokohama, Kanagawa 230-0045, Japan; chunlai.tam@riken.jp (C.T.); akumar@riken.jp (A.K.); 2 Department of Computational Biology and Medical Sciences, Graduate School of Frontier Sciences, The University of Tokyo, Kashiwa, Chiba 277-8561, Japan

**Keywords:** nanobody, single-domain antibody, antibody–antigen complex, pose prediction

## Abstract

Modeling the binding pose of an antibody is a prerequisite to structure-based affinity maturation and design. Without knowing a reliable binding pose, the subsequent structural simulation is largely futile. In this study, we have developed a method of machine learning-guided re-ranking of antigen binding poses of nanobodies, the single-domain antibody which has drawn much interest recently in antibody drug development. We performed a large-scale self-docking experiment of nanobody–antigen complexes. By training a decision tree classifier through mapping a feature set consisting of energy, contact and interface property descriptors to a measure of their docking quality of the refined poses, significant improvement in the median ranking of native-like nanobody poses by was achieved eightfold compared with ClusPro and an established deep 3D CNN classifier of native protein–protein interaction. We further interpreted our model by identifying features that showed relatively important contributions to the prediction performance. This study demonstrated a useful method in improving our current ability in pose prediction of nanobodies.

## 1. Introduction

Knowing the initial binding pose of an antibody (Ab) to its antigen (Ag) is required for in silico Ab affinity maturation and design. There are several approaches which aid the prediction of the Ab binding pose [1]. Being a subclass of protein–protein interaction, one of the main approaches is by protein–protein docking. In some protein–protein docking algorithms, for example, ClusPro [2] and HADDOCK [3], there are dedicated functionalities that improve the prediction of Ab–Ag interaction, such as through incorporating distance constraints of CDR loops in docking. SnugDock, a docking algorithm that improves Ab–Ag complex modeling by sampling CDR loop conformation to account for their flexibility, represents a more specific application to the prediction with the protein–protein docking approach [4]. Apart from predicting the 3D coordinates of Ab–Ag complexes, multiple epitope and paratope prediction methods developed so far [5,6,7,8,9] represent a closely related method class that aids the prediction of Ab–Ag interaction [1]. More recently, there are an emerging number of machine learning models that predict native complex of general protein–protein interaction [10,11,12], with some of them using popular neural network architectures in feature extraction from 3D coordinates of protein–protein complexes, such as 3D convolutional neural networks [13,14] and graph convolutional neural networks [15], which predict the nativeness of protein–protein interactions. These models were able to improve the rankings of native protein–protein complexes from docking and existing re-ranking methods based on interface shape properties [16,17,18,19], evolutionary profile [20], physics and knowledge-based potentials [16,18,19,21,22,23].

Nanobody (Nb) is the variable domain of heavy-chain of the heavy-chain only antibody (VHH) from camelids [24]. Nb possesses several attractive properties as an Ab drug, such as the high thermal stability and the ease of recombinant production in bacterial expression systems [25,26,27,28]. Comprehensive structural characterizations which compare nanobody–antigen (Nb–Ag) and conventional Ab–Ag complexes are available [24,26,27,29]. Compared with conventional antibodies, the incorporation of more of its framework residues in the paratope and the usage of more diverse residue types at the H3 loop for interaction are some of the characteristics of Nb–Ag interactions. Due to these observable differences between nanobodies and conventional antibodies, Nb–Ag interaction represents a characteristic subclass of Ab–Ag interaction. To the best of our knowledge, a prediction tool that is dedicated to the accurate prediction of native Nb poses is currently absent. We reason that Ab pose prediction methods developed so far are presumably suboptimal in predicting native Nb poses because these methods were developed on, and are therefore biased towards, predicting a majority of conventional Ab–Ag and general protein–protein interactions. To make good use of the desirable properties of Nb for *in silico* Ab drug development, there is a need to assess the performance of currently available Ab pose prediction methods on Nb pose prediction and improve their prediction performance.

In this study, we developed an Nb pose prediction model, NbX, and benchmarked its performance with ClusPro [2], which is one of the top performing protein–protein docking method from the latest CAPRI [30] and DOVE [14], a benchmarked binary classifier for native protein–protein interaction through deep 3D convolution. We performed a large-scale self-docking experiment of the available native Nb–Ag complexes with ClusPro. By training a decision tree binary classifier that distinguishes native-like from non-native-like Nb poses with a feature set combining energy, contact and interface property features of the refined parent poses, re-ranking the parent poses using the probability of nativeness showed a significant improvement in the ranking of native-like Nb poses compared with the ranking from DOVE and ClusPro. We further interpreted our model by isolating features that were important in their contribution to the prediction. The Nb pose prediction method introduced in this study serves as a complement to the current Ab pose prediction method in terms of their ability to predict Nb poses. Features that showed importance in distinguishing native-like from non-native-like Nb poses suggest clues to improve our understanding on the interface characteristics of this unique class of single-domain Ab interaction.

## 2. Results and Discussion

### 2.1. Benchmarking with DOVE and ClusPro

**Figure 1:**
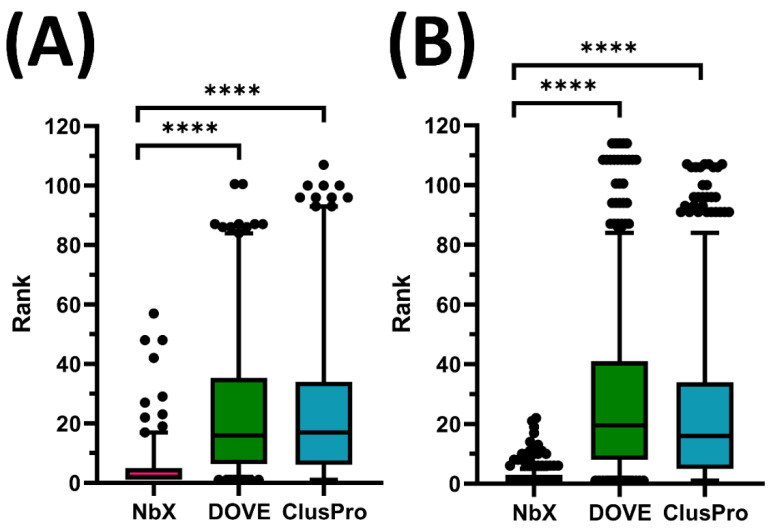
Comparison of re-ranking of native-like Nb pose between NbX, DOVE and ClusPro in (**A**) test set and (**B**) training set. Whole populations (N_test_ = 200 and N_train_ = 660) of ranking of native-like Nb pose from the 5-fold cross-validated were shown in boxplots. The upper and lower whiskers represent 95th and 5th percentile ranking, respectively. The dots represent outliers. Annotations for p value in *t*-Test are as follow, ns: 0.05 < *p* ≤ 1.00; **** *p* ≤ 0.0001.

NbX successfully re-ranked the whole population of native-like Nb poses from the 5-fold cross-validation (N) of the test set (N_test_ = 200) with a significantly higher ranking (*p* < 0.0001) than ClusPro (Figure 1). For test set prediction, the median rank predicted by DOVE and ClusPro were 16th and 17th rank, respectively, while NbX achieved an excellent second rank, demonstrating an eightfold improvement in median ranking. For the 75th percentile ranking, DOVE and ClusPro rankings were beyond 33rd rank while NbX successfully confined the ranking within the fifth rank, or a more than sixfold improvement. For a majority of native-like Nb poses indicated by the 95th percentile, DOVE and ClusPro rankings deteriorated beyond 80th rank while NbX was able to confine their ranking below the 20th, which was a more than fourfold improvement of a majority ranking of native-like Nb pose re-ranked by NbX.

To understand the difference in performance between NbX and DOVE, we further checked the proportion of Nb–Ag complex structures in the training dataset of DOVE. DOVE incorporated one Nb–Ag complex (PDB: 2I25) out of 120 protein–protein complexes in their training set [14]. Apparently, the generalization in predicting native interaction of general protein–protein complexes by DOVE was suboptimal in predicting a specific type of protein–protein interaction, which implied the potential existence of the distinguishable interface characteristics of the Nb–Ag interaction described in previous reviews [26,27,29]. Besides, we note that although the ClusPro ranking was based on cluster size instead of the interface energy of docking decoys, the authors stated that cluster size was roughly proportional to a probability of existence of an energy minimum, which suggested the physical meaning of the ranking by ClusPro [2]. In contrast, apart from only using energy features, NbX attained significantly better re-ranking performance by taking into account the contact and interface property features of Nb–Ag interfaces.

### 2.2. NbX Was Better at Prioritizing Docking Solutions than Determining Absolute Binding Feasibility

The benchmarking of re-ranking performance has demonstrated the ability of NbX in re-prioritizing the docking solutions from ClusPro. To understand NbX further in its prediction ability, we have looked into the performance of NbX in distinguishing native-like and non-native-like Nb poses by the raw native-like probability of native-like poses. Surprisingly, although NbX successfully re-ranked the docking solutions at a significantly better ranking than DOVE and ClusPro, the PR-AUCtest values from the fivefold cross-validation were merely 0.276, 0.205, 0.349 (best), 0.169, and 0.229 (Figure S1), which indicated that NbX was unable to classify native-like poses at high precision and high recall with a single decision threshold of the classification probability. Indeed, by examining the predicted probability of native-like poses of different PDBs from the fivefold cross-validated test sets, we observed a considerable range from 0.00147 to 0.535. However, among refined poses that originated from a single Nb–Ag pair, we reconfirmed that NbX did assign a majority of non-native-like poses with a lower predicted probability of nativeness than the native-like poses, which explained the excellent re-ranking performance of NbX. 

One possible explanation of this phenomenon was the intrinsic hierarchy of the NbX feature set calculated from the refined poses, which were derived from the parent poses from self-docking, leading to a strong clustering in the NbX feature set. Such a hierarchical structure of the feature set potentially allowed efficient learning among poses with considerably similar feature patterns but provided less clues for the learning among poses with distant feature patterns that hindered the meaningful scaling of native-like probability of unrelated Nb–Ag complexes. However, it is worth-noting that the ability in distinguishing native-like from non-native-like poses of a single pair of Nb–Ag was translatable to re-ranking previously unseen Nb–Ag pairs in the test set. Therefore, the current NbX model was more applicable in prioritizing docking solutions of known Nb–Ag interactions than determining the absolute binding feasibility of unrelated nanobodies and antigens with a classification threshold.

### 2.3. Re-Ranking Performance of NbX Was Insensitive to a Substantial Decrease in the Size of the Training Dataset

**Figure 2:**
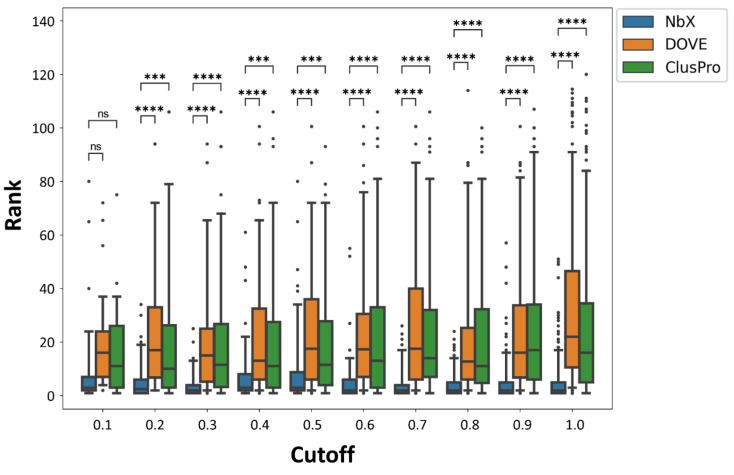
Re-ranking performance of NbX in different cutoffs of pairwise structural alignment quality score. Whole populations of ranking of the native-like nanobody pose from the 5-fold cross-validated test sets were plotted. Annotations for p value in *t*-Test are as follow, ns: 0.05 < *p* ≤ 1.00; ***: 0.0001 < *p* ≤ 0.001; **** *p* ≤ 0.0001.

To minimize information leakage from the training set to the test set, we removed any Nb–Ag complex with the Ag having a pairwise structural alignment score higher than 0.9 to any other Ag from the whole dataset. To assess the robustness of our model when trained with a smaller dataset, we performed a sensitivity test of the prediction performance of NbX to the decrease in the cutoff from 1.0 down to 0.1 (Figure 2). The number of remaining PDBs from different cutoffs were shown in supplementary Table S1. We observed that the re-ranking performance of the NbX model was insensitive to a wide range of the cutoff of structural alignment scores; even down to cutoff = 0.2, the rankings of native-like poses predicted by NbX were consistently higher (*p* ≤ 0.001) than the DOVE and ClusPro ranking. NbX was able to generalize useful patterns of feature distributions from a smaller feature set to improve the re-ranking of native-like Nb poses.

### 2.4. Important Features Contributed to Prediction Performance

In the feature extraction step, in contrast to DOVE, which used deep learned features from 3D coordinates for prediction where the transformations in the latent space were less understandable by humans, NbX represented the conventional feature engineering approach in which the physical meaning of the input features were relatively well-defined. Therefore, there was a good incentive to further interpret the NbX model to understand what were the important features that contributed to the prediction performance of NbX, which may suggest new information that enhances our understanding of the characteristics of the Nb–Ag interface.

**Figure 3:**
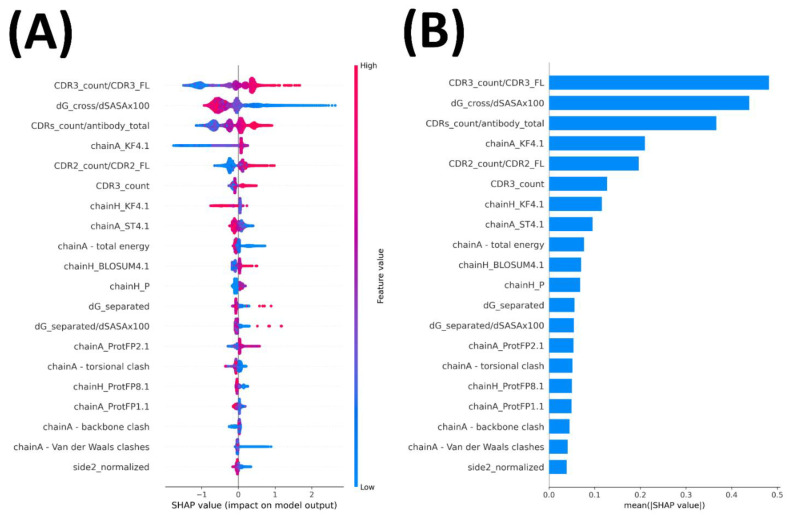
Summary plots of SHAP values of important features contributed to the prediction of NbX in the best single model. (**A**) Scatter plot of SHAP values including directionality of contribution to the predicted probability of nativeness. Each dot represents one prediction in the test set. On the horizontal axis representing the SHAP value, a positive SHAP value represents a positive contribution to the predicted probability and vice versa. The color of dots represents the feature values relative to the maximum and minimum of that feature, which are colored red and blue, respectively. (**B**) Bar plot of mean (|SHAP|) of the important features, which represents the averaged importance of each feature to the test set prediction overall regardless of the directionality of the contribution. ChainA represents Ag and chainH represents Nb.

We calculated the importance measure expressed in the SHAP value of each feature in the test set prediction by the best single model which had the highest PR-AUCtest (Figure 3). The proportion of interacting CDR3 residues in its full length was deemed as the most important feature by NbX, which was consistent with the heavy use of CDR3 for Ag contact in Nbs [26,27]. The energy density feature dG_cross/dSASAx100 calculated from a Rosetta InterfaceAnalyzer was regarded as the second most important feature. An energy dense interface as an important indicator of native-like Nb pose was consistent to the well-known prevalence of energy hotspots in general protein–protein interfaces, including Ab–Ag interactions [31,32,33,34]. The relatively high contact density at the Nb–Ag interface compared with conventional Ab interfaces previously reported [27] highlighted the requirement for energy density in native-like Nb–Ag interfaces.

Apart from the energy and contact features that contributed substantially to the prediction of NbX, several interface property features also showed importance comparable with the energy and the contact features to the prediction of native-like Nb poses by NbX. The hydrophobicity of the epitope and paratope (fourth and seventh most important, respectively) expressed by the kideraFactors [35] contributed the most among all interface property features. Compared with conventional Ab, the higher paratope hydrophobicity of nanobodies was previously reported [26,29]. Representing a different comparison that distinguishes native-like and non-native-like Nb poses through self-docking, NbX tended to classify Nb poses with a less hydrophobic paratope but a more hydrophobic epitope to be native-like (Figure 3a). Following hydrophobicity, we identified the fourth principal component of the ST-scale [36] of the epitope (eighth most important) and the fourth component of the BLOSUM62 substitution matrix of paratope (tenth most important), which has negative correlations to side chain bulkiness, isoelectric point and alpha-helix preference [37], as other important interface property features to the prediction of NbX.

## 3. Materials and Methods

### 3.1. Data Collection and Cleaning

From the SAbDab antibody structure database [38], we collected 371 native Nb–Ag complex structures with protein antigens and a resolution cutoff of 3.5 Å in September 2020. A single biological assembly was isolated from multimeric structures to retain one Nb chain and one Ag chain in each PDB file. Any complex structure with the absence of any of the three CDR loops was removed by checking the CDR conformation database PyIgClassify [39], a total of 260 Nb–Ag complex structures remained (Supplementary data file 1). To remove highly similar Nb–Ag structures which could cause overestimation of prediction performance of the test set, all antigens from the collected complexes were structurally aligned pairwise using superpose from CCP4 [40] to check their structural redundancy. By removing those with the antigens having structural alignment quality score higher than 0.9 with any other antigens, 119 Nb–Ag complexes were retained. 

### 3.2. Rigid-Body Orientation, Backbone and Side Chain Randomization

**Figure 4:**
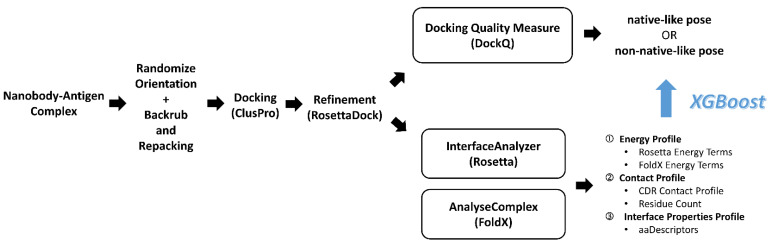
Overall workflow of NbX from data collection to modeling.

We separated the Nb chain and the Ag chain of every Nb–Ag complex into independent PDB files to prepare for docking (Figure 4). Before docking, we randomized the rigid-body orientation and sampled the low-energy backbone dihedral angles and side chain rotamers of all individual Nb and Ag chains. We applied a random translation and rotation in the six axes of freedom to randomize their rigid-body orientation. A RosettaScript performing backrub [41] and side chain repacking was used to optimize the backbone angles and rotamers of side chains, in order to reduce the conformational memories of the interface residues derived from the Nb–Ag complex structures and to make the docking more challenging and realistic. For each Nb and Ag chains, the lowest total energy structure from the 1000 minimized structures was selected for docking.

### 3.3. ClusPro Pose Generation and Refinement

We used the Linux API provided by ClusPro to submit docking jobs of all Nb–Ag pairs to their web server [2]. To assess all available results from ClusPro, we self-docked each Nb–Ag pair with both the default mode and the Ab mode, obtaining 25,658 and 7424 poses, respectively. For each ClusPro pose (considered as the parent pose), a further refinement was performed by RosettaDock consisting of a centroid mode and a subsequent full-atom mode, which provided us a 9.6-fold augmented number of poses for training. After the refinement, a total of 316, 815 refined poses were obtained. 

### 3.4. Pose Classification by Docking Quality Assessment

To classify our refined poses, we used DockQ [42], a benchmarked docking quality score which showed good correlation to the docking quality classes defined by CAPRI [30]. For refined poses with a DockQ score < 0.23, we classified them as “non-native-like” and otherwise “native-like” (Table 1). With this DockQ score cutoff, the “non-native-like” poses were approximately equivalent to “incorrect” poses according to the CAPRI definition [42]. To describe native poses, we used the term “native-like” to indicate a mix of “acceptable”, “medium” and “native” according to the CAPRI definition. This classification divided the refined poses into native-like and non-native-like with 4401 and 312, 414 poses, respectively.

### 3.5. Feature Set Preparation

We prepared a feature set (Table 2) containing a total of 248 features of the refined poses by calculating the energy, contact and interface property profile by InterfaceAnalyzer of Rosetta [43] and AnalyseComplex of FoldX [44]. Apart from the energy terms from the two interface analyzing programs, we calculated CDR contact features, including the proportion of CDR residues in the paratope and the proportion of interface CDR residues versus the full length of each CDR loop, which we hypothesized were useful in guiding the differentiation of native-like and non-native-like poses. To describe the interface properties, we used summations of 66 aaDescriptors [35,36,37,45,46,47,48,49,50,51,52] of the paratope residues and the epitope residues, which served as physicochemical, electrostatic and topological descriptions of the interface.

### 3.6. Model Selection and Training and Test Set Partition

We trained our decision tree model by XGBoost [53] by mapping our energy, contact and interface property features to the binary nativeness classes (Table 3). XGBoost is an implementation of the gradient-boosted decision tree model that demonstrated excellent performance in various classification problems [54,55,56]. In benchmarking, we partitioned 80% (96 PDB IDs) of the Nb–Ag complex to the training set and the remaining 20% (25 PDB IDs) to the test set. The use of the PDB IDs, but not the refined poses, for training/test set partition minimized information leakage from the training set to the test set because refined poses from the same parent pose might contain substantially similar feature values and therefore would lead to overestimation of prediction performance. To account for the randomness of the training/test set partition, which influences the performance due to the random separation of easy and difficult targets, we repeated this partition and the subsequent training and testing 5 times (i.e., 5-fold cross-validated) to evaluate the whole population of test set predictions.

### 3.7. Re-Ranking Method and Benchmarking

We assigned label “1” for native-like poses and “0” for non-native-like poses, our XGBoost model returned a native-like probability as the output. To convert the probability into a ranking, we first calculated the averaged native-like probability of all the refined poses of a parent pose. Then, we sorted the parent poses in descending order of the averaged native-like probability within a single docking run of ClusPro. This re-ranking of parent poses was compared with the ranking from DOVE and ClusPro. DOVE was chosen to further benchmark our model because it represents a similar class of structure-based, machine learning classifiers of protein–protein interactions as NbX. Moreover, DOVE does not use evolutionary information and coordinates of light chains for prediction, which are important to predict the nativeness of Nb–Ag interactions. To generate ranking from DOVE prediction, we ranked the parent poses in descending order of binary probability from the ATOM40 prediction of DOVE. ClusPro was chosen to represent the protein–protein docking method class for benchmarking because of its top performance demonstrated in the latest CAPRI [30] and the compatibility of its Ab mode with Nb–Ag docking, which does not require the existence of the light chain. Ranking from ClusPro was in the order of cluster size of decoys suggested by the ClusPro webserver. To compare the ranking of native-like Nb poses from our model, DOVE and ClusPro, a Wilcoxon matched-pairs signed rank test was performed using GraphPad Prism version 9.1.2 on the population of rankings of all native-like poses generated from the 5-fold cross-validation versus the original ranking from ClusPro and the ranking calculated from DOVE prediction.

### 3.8. Feature Importance Calculation

We used SHAP [57] to calculate the contribution of every feature in the test set predictions. SHAP is a package for isolating important features that contribute most to the prediction performance of machine-learning models. To interpret SHAP value, a positive SHAP value represents a positive contribution to the predicted probability of nativeness and the scalar part of a SHAP value corresponds to the degree of contribution. The ranking of features in descending order of mean (|SHAP|) represents a ranking of importance of the features contributed to the whole test set prediction irrespective of the directionality of contribution.

## 4. Conclusions

In this study, we developed NbX, a machine learning-guided re-ranking method for native-like Nb pose. Through re-ranking with the native-like probability from NbX, we have successfully re-prioritized native-like Nb poses and therefore improved our ability to predict native-like Nb poses through Ab–Ag docking. We demonstrated the usefulness of energy, contact and interface features in describing and distinguishing the interface characteristics of native-like and non-native-like Nb–Ag complexes. The interpretable nature of our NbX model highlighted the describable characteristics of native-like Nb–Ag interactions, which was not offered by the deep learning models in class. Enhanced accuracy of native-like Nb poses prediction will facilitate progress of Nb drug development by *in silico* affinity maturation and design.

## Figures and Tables

**Table 1:** Labeling of native-like and non-native-like poses by DockQ score cutoffs.

CAPRI	DockQ	Labeling
Incorrect	0.00–0.23	Non-native-like (0)
Acceptable	0.23–0.49	Native-like (1)
Medium	0.49–0.80	
High	0.80–1.00	

**Table 2:** Description of individual feature groups of the feature set.

Profile	Feature Group	Number of Features	Description
Contact	Interface residue count	2	Count of residues in paratope and epitope
	Interacting CDR count	3	Count of interacting residues from each CDR loop
	CDR full length	3	Full length of each CDR loop
	Interacting CDR residues in paratope	1	Proportion of total CDR residue in paratope
	Interacting CDR residues versus full length	3	Proportion of CDR residue versus full length of each CDR loop
	Amino acid count	40	Count of individual amino acid in paratope and epitope
Energy	Rosetta InterfaceAnalyzer energy terms	20	Rosetta energy descriptors of the interface
	FoldX AnalyseComplex energy Terms	44	FoldX energy descriptors of paratope and epitope
Interface Properties	Total properties of paratope and epitope	132	Summation of each of 66 aaDescriptors of paratope and epitope

**Table 3:** Summary of settings used for modeling and benchmarking NbX.

Feature Set	Labeling	Partition Sets	Number of PDBs	Model	Classification Type	Validation Method	Ranking Method
Energy, Contact and Interface Properties Profiles	Native-like OR Non-native-like	Training	80% (96 PDBs)	XGBoost	Binary	K-fold Validation (k = 5)	Descending Order of Average Classification Probability of Refined Poses
		Test	20% (25 PDBs)				

## Data Availability

Data is contained within the article and supplementary files.

## Supplementary Materials

The following are available online at https://www.mdpi.com/article/10.3390/ph14100968/s1, Figure S1: The precision-recall curve of (A) training set prediction and (B) test set prediction of the best single model having the highest PR-AUC_test_; Table S1: Performance of NbX in terms of PR-AUC_test_ for different cutoffs of the pairwise structural alignment quality score. The total number of native-like poses in the 5-fold cross-validated test sets was dependent on the random partition of PDB IDs that separated training and test set and thus it did not correlate strictly with the change of the cutoff and the number of PDBs.
