# Supplementary material for: NbX: Machine Learning-Guided Re-Ranking of Nanobody–Antigen Binding Poses
Source: Pharmaceuticals (Basel). 2021 Sep 24;14(10):968. doi: 10.3390/ph14100968 (PMC8537642; doi:10.3390/ph14100968)
Supplement: Supplementary file 1 [file pharmaceuticals-14-00968-s001.zip › pharmaceuticals-1384262-supplementary.pdf]

Supplementary Information

NbX: Machine Learning Guided Re-ranking of Nanobody-Antigen Binding Poses

Chunlai Tam[1,2], Ashutosh Kumar[1], Kam Y. J. Zhang[1,2,*]

[1]Laboratory for Structural Bioinformatics, Center for Biosystems Dynamics Research, RIKEN, 1-7-22 Suehiro, Tsurumi, Yokohama, Kanagawa 230-0045, Japan

[2]Department of Computational Biology and Medical Sciences, Graduate School of Frontier Sciences, The University of Tokyo, Kashiwa, Chiba 277-8561, Japan

*Correspondence should be addressed to Kam Y. J. Zhang (email: kamzhang@riken.jp)

(A)

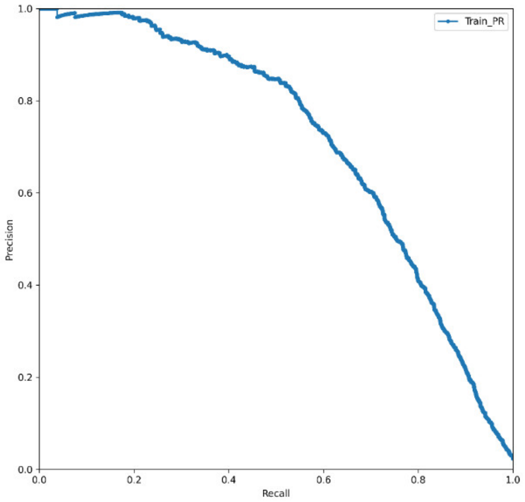

(B)

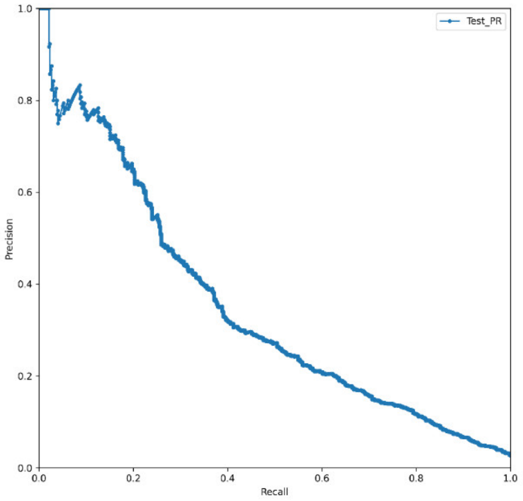

Figure S1: The precision-recall curve of (**A**) training set prediction and (**B**) test set prediction of the best single model having the highest PR-AUC_{test}.

Table S1. Performance of NbX in terms of PR-AUC_{test} for different cutoffs of the pairwise structural alignment quality score. The total number of native-like poses in the 5-fold cross-validated test sets was dependent on the random partition of PDB IDs that separated training and test set and thus it did not correlate strictly with the change of the cutoff and the number of PDBs.

**Cutoff**	**Number of PDBs**	**Number of Native-like Pose_{test}**	**Average PR-AUC_{test}**	**Best PR-AUC_{test}**
0.1	26	47	0.132	0.247
0.2	41	72	0.179	0.358
0.3	49	74	0.225	0.385
0.4	54	79	0.126	0.315
0.5	62	90	0.098	0.166
0.6	70	94	0.185	0.260
0.7	71	97	0.199	0.337
0.8	88	128	0.202	0.268
0.9	119	200	0.246	0.349
1.0	252	407	0.330	0.471
